# Prevalence of DSM-5 diagnostic threshold eating disorders and features amongst Aboriginal and Torres Strait islander peoples (First Australians)

**DOI:** 10.1186/s12888-020-02852-1

**Published:** 2020-09-11

**Authors:** Adam Burt, Haider Mannan, Stephen Touyz, Phillipa Hay

**Affiliations:** 1 School of Medicine, Western Sydney University, Campbelltown, Australia; 2 Translational Health Research Institute (THRI), School of Medicine, Western Sydney University, Campbelltown, Australia; 3 School of Psychology and InsideOut Institute, The University of Sydney, Sydney, Australia; 4 Campbelltown Hospital, SWSLHD, Campbelltown, Australia

**Keywords:** Feeding and eating disorders, Prevalence, Aboriginal and Torres Strait islander, Oceanic ancestry group

## Abstract

**Background:**

There is a dearth of research into mental disorders amongst Aboriginal and Torres Strait Islander peoples (herein First Australians) and especially into eating disorders. In order to understand the healthcare needs of this population, accurate prevalence data is needed. This study aimed to estimate the prevalence of eating disorders amongst First Australians at the diagnostic threshold level and to compare clinical features and health related quality of life (HRQoL) in First and other Australians with and without an eating disorder.

**Methods:**

Data were sourced from the general population 2015 and 2016 Health Omnibus Surveys in South Australia. Trained interviewers conducted via face to face interviews with 6052 people over 15 years old. Eating disorder questions were based on the Eating Disorder Examination and Health Related Quality of Life (HRQoL) measured with the Short-Form 12 v1. The response and participation rates were over 50% and 68% respectively in both surveys. Body Mass Index (BMI) and First Australian status were derived from interview questions. Data were weighted to population norms and analysed using statistical methods for complex surveys.

**Results:**

Twenty-five of 92 (27%) First Australian survey respondents had an eating disorder (majority Other or Unspecified Feeding or Eating Disorder characterised by recurrent binge eating). This was significantly more than the prevalence of other Australians with an eating disorder group (*p* = .04). First Australians with an eating disorder had higher levels of weight/shape overvaluation than all other groups. They were also younger and had poorer Mental HRQoL (MHRQoL) than other Australians without an eating disorder. On logistic regression, First Australian status was not independently associated with having an eating disorder, however, age, Body Mass Index (BMI) and MHRQoL emerged as significant independent variables for the increased rate of eating disorders in First Australians.

**Conclusions:**

Eating disorders were very common in First Australians and were associated with high levels of overvaluation, binge eating frequency and poor MHRQoL. High levels of overvaluation were unexpected. The implications of these findings include an urgent need for further research, and the development of culturally appropriate assessment instruments and treatments for First Australians with eating disorders.

## Background

### Burden and prevalence of eating disorders

According to the Australian Burden of Disease Study, eating disorders accounted for 1.4% of the non-fatal burden of disease in Australia in 2011. They were also the 20th leading cause of Years Lived with Disability (YLD) in females (15,846 YLD); and the 10th leading cause of YLD in 15 to 44 year-old females [1]. Eating disorders are common, with major eating disorders such as anorexia nervosa, bulimia nervosa and binge eating disorder (BED) occurring in 1 in 30 Australians. Other Specified and Unspecified Feeding or Eating Disorders (OSFED and UFED) may be found in 1 in 30 and 1 in 10 Australians respectively [2, 3].

### Prevalence of eating disorders amongst First Australians

A recent systematic review into the prevalence of mental disorders amongst First Australians did not identify any studies addressing the prevalence of diagnosed eating disorders amongst First Australians [4]. Only a few studies have presented data on disordered eating and eating disorder symptoms in First Australians. A cross-sectional study in South Australia aimed to establish the 3-month prevalence of eating disorder behaviours (binge eating, restrictive dieting, purging, and core eating disorder psychopathology). The authors suggested that compared to the non-Indigenous population, a relatively higher proportion of First Australians reported binge eating, subjective binge eating and purging behaviours. These data were limited by the sample size (total *n* = 3047, First-Australians *n* = 85) and that eating disorders to the diagnostic threshold level were not assessed [5]. A systematic synthesis of population health data in adolescent First Australians suggested eating disorders were more likely to affect non-Indigenous Australians compared to First Australians, and were more likely to affect First Australians in urban areas compared to remote areas, as no eating disorder cases were identified in remote areas [6]. Data from a cross-sectional survey of 6041 people in South Australia addressed socioeconomic correlates of eating disorders. The authors found those who indicated they were First Australians (Indigenous) had similar levels of eating disorder features to non-Indigenous people, however, this study did not assess eating disorders to the diagnostic threshold level [7].

### Mental health of First Australians

It is important to investigate eating disorders in First Australians. Their health is known to be poor and in one survey 12% of First Australians reported feeling depressed, or suffering depression as a long-term health condition, and 30% reported high to very high levels of psychological distress, which is 3-fold higher than the non-Indigenous population [8]. Another study of over 34, 000 people found First Australians in Victoria were more likely to suffer psychological distress, based on a Kessler scale score greater than 22, compared to non-Indigenous Victorians (OR 2.56; 95% (CI): 1.67–3.93). The study found low socioeconomic status, poor perception of the neighbourhood, inability to get help from family, and social and civic distrust largely explained the higher prevalence of psychological distress among First Australian adults in Victoria [9]. Data from the Australian Burden of Disease Study suggests mental illnesses make up the greatest burden of non-fatal disease, in Years Lived with Disability (YLD) in First Australians. The top three causes of disease burden (percent of total YLD) in First Australians were Anxiety Disorders (9.4%), Alcohol Use Disorders (8%) and Depressive Disorders (7.2%). In First Australian men, the greatest burden was due to Alcohol use disorders, depressive and anxiety disorders; in First Australian women the greatest contributors were anxiety and depressive disorders, musculoskeletal and alcohol use disorders [10]. All of these conditions are commonly comorbid with eating disorders [11].

### Gaps and aim

In order to understand the burden and health care needs of First Australians general population prevalence estimates are needed. Based on the previous research, we hypothesised that eating disorders may be as common, if not more common in First compared to other Australians, but to our knowledge there had been no previous research establishing the diagnostic threshold prevalence of eating disorders amongst First Australians. This study thus aims to estimate the DSM-5 diagnostic threshold prevalence of eating disorders amongst First Australians and to measure the Mental Health Related Quality of Life (MHRQoL) in First Australians compared to other Australians with an eating disorder.

## Methods

In this study we sourced data on First Australian identification and Eating Disorder features taken from the Health Omnibus Survey (HOS) 2015 and 2016. For this and other similar cross-sectional studies of this data we pooled data from each year to create one data set. Data for the HOS are collected by Harrison Health Research in South Australia, and it is a comprehensive survey of households in South Australia, both rural and urban providing detailed health information for community and medical research in Australia.

### Participant selection

The methods for the 2015 and 2016 surveys were similar. The study was conducted in South Australia (SA); the regions of SA were separated by their Statistical-Area Level 1 (SA1) classification and selected based on Probability Proportion to Size and Australian Bureau of Statistics 2011 Census reference data. Three thousand and ninety-eight households were identified in metropolitan areas, and all rural centres with a population over 10,000 were selected. Some towns with populations over 1000 were selected based on probability proportion to size. Individual households were selected based on a procedure of randomly selecting one household, then selecting every fourth household until 10 households from that SA1 had been selected. One interview was conducted at each selected household with a resident over the age of 15 years. The person invited to conduct the survey in a house of more than one person over 15 years was the one with the most recent birthday.

A pilot study of 50 interviews was conducted before undertaking the study. Ten-per cent of households in 2015 and 2016 who participated in the study were contacted by the Operations Manager of Harrisons Health Research to ensure the survey was conducted, and then the person with the most recent birthday (at the time of interview) was the one who participated in the study. There was a 53.7% (*n* = 3005) and 58.4% (*n* = 3047) response rate in 2015 and 2016 respectively (the per cent of interviews conducted based on the number of households selected). The participation rates (proportion of interview based on eligible individuals) were 71.1 and 68% respectively. Reasons for non-response in both surveys included: refusal *n* = 1129, contact unable to be established after 6 visits at differing times *n* = 736, non-English speaking respondent *n* = 70, selected respondent away for duration of the survey *n* = 57, Illness/mental incapacity *n* = 99, locked gate/unable to access property n = 73, ferocious dog n = 7, terminated interview no explanation *n* = 2.

### Ethics

Adult participants provided verbal rather than written informed consent, due to the practicalities of carrying out a large-scale survey and the low risk nature of the survey content. For minors (15–17 year olds) enrolled in the study, written consent was obtained from the participant’s parent/guardian. The surveys and methods of obtaining consent were approved by the University of Adelaide Human Ethics Committee H-097-2010.

### Interviews

Structured, respondent-based interviews where administered by trained interviewers covering health and demographic questions. Body Mass Index (kg/m2) was calculated based on self-reported weight and height. Eating Disorder behaviours were assessed based on questions adapted from the Eating Disorder Examination instrument in Fairburn, 2008 [12].

#### Questions on eating disorders included

- Purging behaviours. Participants were invited to answer “yes”, “no” or “refused to answer”*.*

*Over the past three months have you regularly used, that is at least once a week, any of the following: laxatives, diuretics (water tablets), made yourself sick, in order to control your shape or weight?*

- Binge eating. The interviewers noted whether the participants reported that they were unable to stop eating once they started or could not prevent themselves from overeating. Participants were invited to answer, “not at all”, “less than weekly”, “once a week”, “two or more times a week”, “don’t know”, or to “refused to answer”.

*I would now like to ask you about episodes of overeating. By overeating, or binge eating, I mean eating an unusually large amount of food in one go and at the time feeling that your eating was out of control. Over the past three months how often have you overeaten?*

- Overvaluation of weight and shape. Participants were invited to answer based on a seven-point scale, 0 “being not at all important” and 6 being “extremely” or “the most important issue”.

*How important has your weight and/or your shape been to how you think about (judge or view) yourself as a person in the past three months?*

- Very strict dieting and fasting with aim of controlling body shape and weight. Participants were invited to answer “yes”, “no” or “refused to answer” two questions about very strict dieting.

*Over the past three months have you regularly done any of the following: gone on a very strict diet, or eaten hardly anything at all for a time, in order to control your shape or weight?*

*Are you currently avoiding or restricting eating any foods to the degree that you have lost a lot of weight and/or become lacking in nutrition (e.g.have low iron) and/or had problems with family, friends or at work?*

- Avoidant Restrictive Food Intake Disorder (ARFID) was assessed with the questions:

*Are you currently avoiding or restricting eating any foods to the degree that you have lost a lot of weight and/or become lacking in nutrition (e.g., have low iron) and/or had problems with family, friends or at work? for cultural or medical reasons e.g., Lent, Ramadan, nut or other food allergy.*

*Possible answers recorded in the affirmative:*

*“Yes” - ‘dieting’ to prevent weight gain; and.*

*“Yes” - any other reason e.g., food dislike or fear of swallowing.*

#### First Australian status

This was asked with the following question*: Are you of Aboriginal or Torres Strait Islander origin?* with five exclusive responses of: “no”, “Aboriginal”, “Torres Strait Islander”, “both”, or “don’t know”.

#### Health related quality of life

Current (past four weeks) mental and physical health related quality of life (MHRQoL and PHRQoL) were assessed with the Short-Form-12 item questionnaire v1 [13]. MHRQoL and PHRQoL summary scores range from 0 to 100 with a lower score indicating poorer HRQoL. It has been widely used to measure HRQoL in a large number of medical and psychiatric disorders and has robust psychometric properties [2]. Internal consistency Cronbach’s α in the 2016 survey was 0.85, 0.82 for PHRQoL component scores and 0.76 for MHRQoL component scores [14]. (Raw score data were not provided to the authors to calculate this in the 2015 survey).

For the purpose of this study ICD-11 [15] criteria are applied which in this study also were comparable to the DSM-5 [16] with the exception that the Criterion B diagnostic specifiers are not required for binge eating disorder (BED) in ICD-11. Anorexia nervosa broad (AN-B) was diagnosed where the criteria of self-maintained underweight status (DSM-5 Criterion A) with weight/shape overvaluation (DSM-5 Criterion C) were met. Full definitions are found in Hay et al. (2017) [3].

#### Statistical analysis

Data were inspected and cleaned. On data inspection, 1671 participants did not report their First Australian status. These data were treated as missing in this study and were estimated before performing all statistical analysis using multiple imputation which is the state-of-the-art method to handle missing data [17]. Twenty-five imputations were performed. SAS 9.4 was used to perform all data analysis. All descriptive statistics, with the exception of eating disorder diagnostic types, reported in this study were weighted to population norms after taking into account complex study design due to stratified cluster random sampling. (Diagnostic types are presented using unweighted data because of very small numbers in each group.) The association between two categorical variables was examined using WLS score statistic for complex survey data [18] as it always exists while the commonly used Rao-Scott statistic could not be estimated in our study due to one or more cells having zero count. Differences between groups for a continuous outcome having normal distribution was tested with one-way ANOVA for complex surveys (using SAS PROC SURVEYREG) and if the global F test was significant, Tukey-Cramer post-hoc tests were performed for multiple comparisons. Although for simple surveys where data are collected using simple random sampling, Kruskall Wallis with Mann-Whitney U or Wilcoxon sum rank post-hoc tests are commonly used for comparing more than two groups for a non-normal outcome, these are not available for complex surveys when the independent variable has more than two categories [19]. So, instead, Moody’s median test for complex surveys was used to test for any difference between the four groups and if found significant median post-hoc tests with Bonferroni adjustments were performed for multiple comparisons [20]. An advantage of median test over Wilcoxon sum rank or Mann Whitney U test is that it only tests for differences in the median irrespective of any differences in the shape of the distribution [20]. Finally, to determine the significance of independent variables for presence of any eating disorder, survey logistic regression (using SAS PROC SURVEYLOGISTIC) was performed. The variables examined included age, BMI, sex, MHRQoL, PHRQoL and First Australian status while their odds ratios with respective 95% confidence intervals were reported. A significance level of .05 was employed for testing the significance of all chosen variables. The results of all statistical analyses were pooled using Rubin’s rules [21]. In order to examine the question that First Australian status is also non-significant if there is complete case analysis without imputing the missing data, we further conducted a sensitivity analysis to see if the data missing for first Australians were missing not at random or not. This was according to the method of Sterne et al. (2009) [22] who stated that,” Therefore, biases caused by data that are missing not at random can be addressed only by sensitivity analyses examining the effect of different assumptions about the missing data mechanism”.

## Results

Twenty-five (weighted data) of 92 (27%; 95%CI 19.1–37.0) First Australian respondents had an eating disorder, which was significantly more than the other Australian groups (χ2 = 4.25, df = 1, p .039). Using unweighted data, 23 First Australians had an eating disorder: 3 had BN, 1 had Binge Eating Disorder (BED), 2 had AN-Broad, 1 had OSFED purging disorder, 2 had OSFED Atypical AN, 1 had ARFID and a further 13 had UFED (all with recurrent binge eating without marked distress). Fifty-three (58%) First Australians were women and there were no significant differences in the proportions of First Australian men and women with and without an eating disorder (χ2 = 1.226, df = 1, *p* = .343, Fisher Exact test).

As shown on Table 1 there were no significant differences between the First Australians with and without an eating disorder in age, BMI, MHRQoL and PHRQoL. First Australians with or without an eating disorder were significantly younger and had higher levels of overvaluation than other Australians without an eating disorder. First Australians with an eating disorder and Other Australians with an eating disorder had a similarly high frequency of Binge eating, and significantly higher frequency of binge eating than First and Other Australians without an eating disorder. They also had poorer MHRQoL than any other group but this did not reach significance.

**Table 1:** Comparative features of First Australians (FA) and other Australians (OA) with and without an Eating Disorder (ED)

	FA with ED (A)	FA without ED (B)	OA with ED (C)	OA without ED (D)	Statistic	***P***	Post-hoc^**I**^
*N*	**25**	**68**	**1071**	**4889**			
**Mean (SE)**					**ANOVA F (df)**		
**Age (years)**	36.49 (3.59)	37.54 (3.26)	39.98 (2.03)	48.48 (2.42)	66.50 (36048)	<.0001	A,B, C ≠ D
**BMI (kg/m**^**2**^**)**	29.10 (1.43)	27.45 (0.91)	28.52 (0.20)	26.66 (0.19)	32.28 (36048)	<.0001	D ≠ C
**Physical HRQoL**	45.64 (3.15)	47.50 (2.13)	48.94 (0.57)	48.81 (0.60)	1.25 (36048)	.29	n.a.
**Mental HRQoL**	48.47 (2.37)	49.44 (2.62)	50.06 (0.54)	52.87 (0.24)	34.29 (36048)	<.0001	C ≠ D
**Median (IQR)**					**Moody’s Median (df)**		
**Binge eating**^**III**^	2.46 (1–3.15)	1.00 (1–1)	2.58 (2–3.22)	0.93 (0.89–0.98)	2119.71 (3)	<.0001	A ≠ B,D B ≠ C, C ≠ D
**Over-valuation**^**III**^	4.43 (2.44–5.39)	3.72 (1.61–4.67)	3.39 (2–4.56)	2.76 (0.64–3.87)	232.75 (3)	<.0001	A,B,C ≠ D
***n*** **(%)**					χ^2^ **F (df1,df2)**		
**Purging**	1 (4.00%)	0 (0%))	51 (4.76%)	0 (0%)	3.05 (3,11)	0.074	n.a.
**Diet/fasting**	6 (24%)	0 (0%)	265 (24.74%)	20 (.40%)	2.17 (3,11)	0.150	n.a.

^I^The post-hoc test used for pairwise comparison of means was the Tukey-Cramer statistic and Bonferroni-corrected pairwise Median statistic was used post-hoc Moody’s median test. ^II^Binge eating 1 = none, 2 = <weekly, 3 = weekly, 4 = > weekly episodes; ^III^Overvaluation 0 being not at all important and 6 being extremely or the most important issue; n.a. indicates not applicable as Rao-Scott chi-squared test cannot be performed because at least one cell has zero frequency. *ED* Eating Disorder, *BMI* Body Mass Index, *HRQoL* Health Related Quality of Life, *df* degrees of freedom, *χ*^*2*^ Chi Squared test, *n* number of participants

As shown on Table 2, in the logistic regression analysis age, BMI and MHRQoL emerged as significant independent variables for the increased rate of eating disorders in First Australians. Sex and First Australian status were not retained as independent variables in the model.

**Table 2:** Results of logistic regression with dependant variable- presence of an eating disorder

	Exp (B)	95% CI	***P*** value
**Age (years)**	0.973	0.970; 0.976	< .0001
**BMI (kg/m2)**	1.065	1.049; 1.081	< .0001
**Mental HRQoL**	0.973	0.967; 0.979	< .0001
**Sex**	1.068	0.947; 1.203	0.284
**First Australian status**	1.082	0.894; 1.309	0.421

*BMI* body mass index, *HRQoL* Health Related Quality of Life

### Sensitivity analysis

We found that based on complete case analysis (following casewise deletion of missing data) the odds ratio for the effect of first Australians on ED was 1.227, *p* = 0.2697, 95% CI (0.837, 1.798). We also found that this effect was also non-significant based on multiply imputed data assuming missing at random (OR = 1.082, *p* = 0.421, 95% CI (0.894, 1.309). Based on the above, there was an underestimation of the odds ratio by 11.82% when missing data for first Australians are imputed compared to being casewise deleted. In such situation, the missing data in the first Australian variable is systematic and we can’t rule out missing not at random. There was insufficient data to estimate the odds ratio correctly assuming missing not at random and hence the degree of bias associated with missing not at random cannot be estimated.

## Discussion

This study is the first to our knowledge to estimate the diagnostic threshold prevalence of eating disorders in a general population sample of First Australian adults. It supports earlier Australian research [5, 6] in finding eating disorders were common in First Australians. With the exception of UFED characterised by recurrent binge eating no one eating disorder appeared to be more common than others. (The high numbers of UFED with binge eating without marked distress has been observed previously. This is a poorly defined group and may reflect a spectrum of illness that may cross over into subclinical syndromes [3].) Sex and First Australian status were not retained as significant independent variables for eating disorders, however, age, BMI and MHRQoL were retained in the model. As First Australians were younger, had a higher BMI and had poorer Mental HRQoL, it is likely these are the reason for the high numbers of eating disorders within this population. It is unclear why First Australians with an eating disorder did not differ from First Australians without an eating disorder on BMI, MHRQoL and PHRQoL, and further research is needed to explain this**.** However, it is possible the present study lacked power to detect such differences.

There is very little published research on eating disorders amongst First Australians, therefore there is very little to inform practice [4]. First Australians are the most disadvantaged group in Australia and experience numerous barriers to even the most basic forms of healthcare [8]. First Australians are also a culturally and linguistically distinct group with very different healthcare needs. Therefore, this paper provides important prevalence data to clinicians, and an impetus to the need to screen for these conditions. In particular, together with other health services, Aboriginal Community Controlled Health Services (ACCHS) and Aboriginal Medical Services (AMS) have an important role in conducting Medicare Health Assessments for Aboriginal and Torres Strait Islander People (Medicare Benefits Schedule item 715). This research highlights the need to screen and assess for eating disorders in First Australians during these health assessments. There is also a need to increase awareness of eating disorders in First Australians amongst primary care providers and a need to develop culturally informed mental health services and assessment instruments to manage these eating disorders in First Australians. An exemplar of the latter is the adapted nine-item Patient Health Questionnaire (aPHQ-9), a validated and culturally specific screening tool for depression in First Australians [23].

The missing data and small numbers of First Australians were notable limitations of this research. The estimated statistical error of the findings is however in favour of the null hypothesis so that the size of the problem of EDs in First Australians that we report is a conservative estimate in the direction of underestimating the true prevalence. The numbers of participants were consequently too small to investigate single eating disorder types within the population. There is a need to further investigate eating disorders in a larger sample of First Australian individuals and in adolescents and to develop culturally informed assessment instruments. Due to the design, this study cannot demonstrate causation of eating disorders.

One thousand six hundred and seventy-one participants did not answer the question on First Australian status in the survey. This group is likely to include First Australians.

First Australians are the most disadvantaged group in Australia which is contributed to by a history of colonisation, genocide and issues such as not being counted as *people* in the census until the 1971. Therefore, First Australians may be reluctant to disclose their First Australians status in an official capacity, especially to non-Indigenous people or any official type of person, such a census or survey taker. Fear of government, feelings of shame, fear of judgement, identity confusion or greater identification with another ethnic group are amongst the reasons one may not close their First Australian status. In this study, First Australians had higher levels of overvaluation of weight and shape; previous research using HOS data suggested those who declined to answer questions on First Australian status were also more likely to experience overvaluation of weight and shape [7]. These data were treated as missing in this study. However, it is possible the estimates of eating disorders in First Australians would have been even higher if these participants had provided their status.

The study had several strengths. It was a representative general population survey. The use of a structured interview by trained and supervised interviewers versus self-report questionnaires allowed the exploration of responses and improved the quality of assessment and accuracy of the estimates of diagnostic threshold prevalence in the First Australian population. However, limitations were that weight and height were provided by self-report and diagnoses were not confirmed by a clinical interview.

In future research there is a need to develop culturally appropriate assessment instruments and treatments for First Australians. This study should be repeated in larger samples and in younger people; we suggest in areas with large First Australian populations such as Western Sydney and the Central Coast of New South Wales. There is also a need for qualitative research to deepen our understanding of eating disorders in First Australians, which will assist in the development of assessment instruments.

## Conclusions

Based on our findings, eating disorders are common in First Australians and are associated with high levels of overvaluation of weight and shape and binge eating. The high levels of overvaluation were an unexpected finding. There is an urgent need for further research in a larger population of First Australians to confirm these findings, and to assist in the development of culturally appropriate assessment instruments to detect eating disorders in First Australians. Eating disorders are also likely occur in the context of other indices of health status such as a high BMI and poorer MHRQoL. Thus, we suggest General Practitioners and others who see First Australian patients should screen for and consider eating disorders during physical and mental health assessments.

## Data Availability

Data are available from the corresponding author for the purpose of secondary data analyses.

## Abbreviations

ED: Eating Disorder
BMI: Body Mass Index
HRQoL: Health Related Quality of Life
df: Degrees of freedom
χ^2^: Chi Squared test
MWU: Mann Whitney U test
n: Number of participants
YLD: Years Lived with Disability
HRQoL: Health Related Quality of Life
MHRQoL: Mental Health Related Quality of Life
PHRQoL: Physical Health Related Quality of Life
BED: Binge Eating Disorder
AN-B: Anorexia Nervosa Broad
ARFID: Avoidant Restrictive Food Intake Disorder
BN: Bulimia Nervosa
OSFED: Other Specified Feeding or Eating Disorder
UFED: Unspecified Feeding or Eating Disorder
aPHQ-9: Adapted Patient Health Questionnaire
AMS: Aboriginal Medical Service
ACCHS: Aboriginal Community Controlled Health Service
ICD-11: International Classification of Disease 11th revision
DSM-5: Diagnostic and Statistical Manual of Mental Disorders 5th edition
HOS: Health Omnibus Survey
